# A Study on the Potential of Valorizing *Sargassum latifolium* into Biofuels and Sustainable Value-Added Products

**DOI:** 10.1155/2024/5184399

**Published:** 2024-09-10

**Authors:** Nour Sh. El-Gendy, Mohamed Hosny, Abdallah R. Ismail, Ahmad A. Radwan, Basma A. Ali, Hager R. Ali, Radwa A. El-Salamony, Khaled M. Abdelsalam, Manal Mubarak

**Affiliations:** ^1^Egyptian Petroleum Research Institute (EPRI), Nasr City, P.O. 11727, Egypt; ^2^Center of Excellence, October University for Modern Sciences and Arts (MSA), 6th of October City, P.O. 12566, Egypt; ^3^National Research Centre (NRC), El-Dokki, Cairo, P.O. 12622, Egypt; ^4^General Organization for Export and Import Control (GOEIC), Cairo, Egypt; ^5^Marine Environment Division, National Institute of Oceanography and Fisheries NIOF, Alexandria Branch, P.O. 21519, Egypt; ^6^Soil and Water Department, Faculty of Agriculture, Ain Shams University, Cairo, P.O. 11241, Egypt

## Abstract

To increase the limited commercial utility and lessen the negative environmental effects of the massive growth of brown macroalgae, this work illustrates the feasibility of valorizing the invasively proliferated *Sargassum latifolium* into different value-added products. The proximate analysis recommends its applicability as a solid biofuel with a sufficient calorific value (14.82 ± 0.5 MJ/kg). It contains 6.00 ± 0.07% N + P_2_O_5_ + K_2_O and 29.61 ± 0.05% organic C. Its nutritional analysis proved notable carbohydrate, ash, protein, and fiber contents with a rational amount of lipid and a considerable amount of beneficial macronutrients and micronutrients, with a low concentration of undesirable heavy metals. That recommends its application in the organic fertilizer, food, medicine, and animal fodder industries. A proposed eco-friendly sequential integrated process valorized its biomass into 77.6 ± 0.5 mg/g chlorophyll, 180 ± 0.5 mg/g carotenoids, 5.86 ± 0.5 mg/g fucoxanthin, 0.93 ± 0.5 mg/g *β*-carotene, 21.97 ± 0.5% (w/w) alginate, and 16.40 ± 0.5% (w/w) cellulose, with different industrial and bioprocess applications. Furthermore, *Aspergillus galapagensis* SBWF1, *Mucor hiemalis* SBWF2, and *Penicillium oxalicum* SBWF3 (GenBank accession numbers OR636487, OR636488, and OR636489) have been isolated from its fresh biomass. Those showed wide versatility for hydrolyzing and saccharifying its polysaccharides. A Gram-negative *Stutzerimonas stutzeri* SBB1(GenBank accession number OR764547) has also been isolated with good capabilities to ferment the produced pentoses, hexoses, and mannitol from the fungal saccharification, yielding 0.25 ± 0.014, 0.26 ± 0.018, and 0.37 ± 0.020 g ethanol/g algal biomass, respectively. Furthermore, in a pioneering step for valuing the suggested sequential biomass hydrolysis and bioethanol fermentation processes, the spent waste *S. latifolium* disposed of from the saccharification process has been valorized into C-dots with potent biocidal activity against pathogenic microorganisms.

## 1. Introduction

Climate change and its concomitant issues, such as rising seawater temperatures, have resulted in the progressive growth of brown macroalgae and their massive spread across the Red Sea shorelines [1]. *Sargassum* sp. is considered one of the main brown macroalgae that inhabit the Red Sea [2–4]. Overfishing activities, increased levels of nutrients and organic pollutants due to the open discharge of industrial effluents, and other activities related to the oil and gas industry contribute to the overgrowth of brown macroalgae [5]. The massive growth of brown algae leads to eutrophication problems, reducing light penetration, and negatively affecting biodiversity and life underneath water [6]. Its multi-increment antagonistically competes with coral reefs, suffocating reefs, which accelerates reef degradation and causes coral bleaching and death [7]. Their massive growth also has the potential to entangle marine mammals and turtles [8]. Not only that, but their presence along shorelines also has a detrimental effect on turtle nesting [9]. In addition to creating issues with solid waste management, macroalgal blooming also produces hydrogen sulfide gas, which is poisonous and corrosive, creates piles on shorelines that may decay, forming a bad smell, and produces methane that rigorously adds to the problem of climate change [10] and leads to the invasion of insects that can irritate the skin [11]. Consequently, this causes harm to shorelines and has severe negative effects on aquaculture, the food chain, the marine ecosystem, tourism, and the economy [6].

Because of fast-growing globalization and industrialization, besides the worldwide exponential growth of the human population, the demand for different industrial feedstock, fertilizers, animal fodders, and energy has increased. Recently, among the sustainable resources, marine macroalgae have attracted researchers and the industrial sector as being a promising, readily available, and cost-effective feedstock for different industrial applications. They do not cause the food versus fuel problem; they do not require fertilizer, pesticides, fresh water, arable land, or farming lands for cultivation [12]. Because they grow quickly and produce a lot of biomass per area, and because they have low amounts of lignin and high amounts of carbohydrates, they show a lot of promise for use in biorefineries and the production of biofuels [13].

The utilization of *Sargassum* biomass as a feedstock in biorefineries for the production of various industrial value-added products, such as platform chemicals, animal feeders, and biofertilizers, besides third-generation biofuels, has recently attracted increased industrial interest [14].

In an attempt to overcome that issue and substantiate the perception that waste is not a waste until it is wasted, this work investigates the feasibility of *Sargassum latifolium* acting as a habitat for microorganisms capable of hydrolyzing, saccharifying, and fermenting its lignocellulosic biomass and carbohydrate constituents into fermentable sugars and bioethanol. It also studies the applicability of *Sargassum latifolium* as a precursor for organic fertilizer, animal fodder, primary solid biofuel, and C-dot with considerable biocidal activity against pathogenic microorganisms. Furthermore, it suggests an eco-friendly, fully integrated process for its valorization into pigments, antioxidants, alginate, and cellulose with various industrial applications.

## 2. Materials and Methods

### 2.1. Sampling of Macroalgal Biomass and Its Associated Water

**Figure 1:**
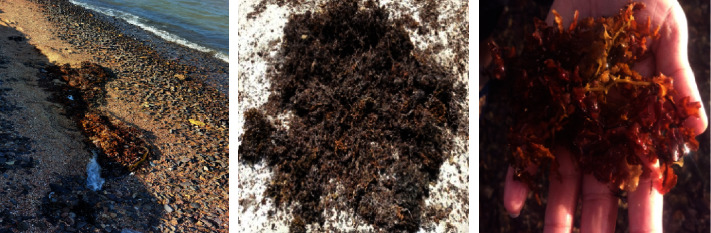
*Sargassum latifolium* on Egyptian Red Sea shoreline (27°23′11″N 33°47′17″E).

The brown *Sargassum latifolium* biomass and its associated water sample have been collected from Hurghada city shoreline, Red Sea Governorate, Egypt, during the autumn season of 2022 (27°23′11″N 33°47′17″E) (Figure 1).

The microscopic examination of the collected *Sargassum latifolium* has been performed according to Liang et al. [15] and Yeh et al. [16] using a stereo microscope (Novex, model P-20, Euromex microscopes BV, Arnheim, the Netherlands) and a light microscope (Optika model B-192, S.r.l.Via Rigla, Ponteranica (BG), Italy).

Water analysis for the collected algal biomass-associated water was performed in the Egyptian Petroleum Research Institute's Central Analytical Labs, according to ASTM [17]. Anions and cations were determined according to ASTM D4327 [17] and ASTM D6919 [17] using ion chromatography (Thermo-Dionex™ ICS-6000 Capillary HPIC™ System equipped with high capacity columns AS9 and CS12 for anion and cations, respectively, Thermo Fisher Scientific, Carlsbad, California, USA). Heavy metals were determined according to ASTM D4691 [17] using a Flame Atomic Absorption Spectrophotometer (model ZEEnit 700 P, Analytik Jena GmbH, Hena, Germany). Total dissolved solids (TDS) and total suspended solids (TSS) were determined according to ASTM D5907 [17] for filterable matter (TDS) and nonfilterable matter (TSS) in water. Conductivity and resistivity were determined on-site according to ASTM D1125 [17] using a digital conductivity meter (model WTW 330i, Willis Towers Watson Public Limited Company, London, UK). Density and specific gravity were determined according to ASTM D1429 [17]. pH was determined according to ASTM D1293 [17] using a digital pH meter (model Mettler Toledo-Seven GoTM, Ohio, USA). Alkaline species (CO_3_, OH, and HCO_3_) were measured according to ASTM D3875 [17]. Alkalinity was determined according to ASTM D1067 [17]. The hardness value of water (Ca and Mg) was determined according to ASTM D6919 and calculated according to ASTM D1126 [17]. Biochemical oxygen demand (BOD) and chemical oxygen demand (COD) were measured according to APHA method 5210 [18] and ASTM D1252 [17], respectively. Salinity was calculated according to APHA method 2520 [18].

### 2.2. Macroalgal Biomass Preparation

After being gathered, the macroalgae were cleaned with tap water to remove any remaining dirt, salt, and sand. They were then left to dry in the sun. After that, they were homogenized into a 0.8–1 mm powder by grinding and sieving. Lastly, they were stored at room temperature until needed in clean, sealed plastic bags.

### 2.3. Physicochemical Characterization of the Collected Algal Biomass

The nutritional components of *S. latifolium* biomass were determined according to the techniques outlined in AOAC [19]. The proximate analysis, which involved measuring the calorific value [20], volatile matter [21], ash [22], and moisture [23] contents, has also been done. The method described by Moubasher et al. [24] has been used to determine the amounts of cellulose, hemicellulose, and lignin in the collected *S. latifolium* biomass. The biochemical analysis was conducted according to El-Gendy et al. [25]. The Walkley and Black method [26] was used to determine the concentrations of organic carbon and matter. The Jackson [27] method was used for measuring the electrical conductivity and pH. The Kjeldahl digestion method, as reported by Chapman and Pratt [28], was utilized to quantify total N, and the crude protein concentration was computed using the conversion factor (6.25). The method of Watanabe and Olsen [29] was used to quantify total phosphorus. ICP-MS (inductively coupled plasma mass spectrometry, Spectro Ciros CCD ICP-OES, Spectro Analytical Instruments, Kleve, Germany) was used in accordance with the methodology outlined by Benton [30] to determine the minerals and heavy metal contents.

### 2.4. A Fully Integrated Process for Extraction of Various Value-Added Products from *S. latifolium* Biomass

**Figure 2:**
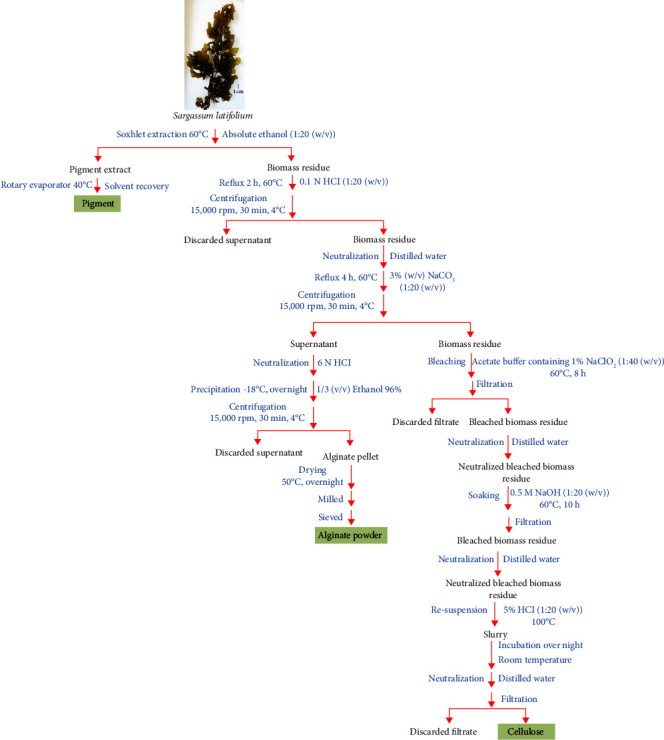
A fully integrated process for the production of value-added products from the collected *S*. *latifolium*.

The pigments and alginate were sequentially extracted from the algal biomass according to the method reported by Trica et al. [31], and then the cellulose was extracted from the leftover biomass according to the method reported by Trivedi et al. [32].  Figure 2 shows the whole integrated process that is sequentially used to extract various value-added products.

The extracted pigments were detected by scanning the distinctive absorption spectra at wavelengths of 190–800 nm using a UV/Vis/NIR double beam spectrophotometer (model V-570, JASCO International Co., Ltd., Tokyo, Japan) [33].

According to Ismail [33], the extracted concentration of chlorophyll a and *β*-carotene was calculated as follows;

(1) $$\begin{array}{c} \text{Total Chloropyll }\left(\text{mg}/g\right)=\frac{\left[20.2A_{645}+8.02A_{663}\right]\times V}{W}, \end{array}$$

(2) $$\begin{array}{c} \text{Carotenoids}\left(\text{mg}/g\right)=\frac{{4A}_{480}\times V}{W}. \end{array}$$

The preliminary calculation of fucoxanthin concentration was done according to Ktari et al. [34]:

(3) $$\begin{array}{c} \text{Fucoxanthin }\left(\text{mg}/g\right)=\frac{\left[7.69\times \left(A_{480}-\;A_{750}\right)-5.55\times \left[\left(A_{631}-A_{750}\right)+\left(A_{582}-A_{750}\right)-0.297\times \left(A_{665}-A_{750}\right)\right]-0.377\times \left(A_{665}-A_{750}\right)\right]\times V}{W}. \end{array}$$

The preliminary calculation of *β*-carotene concentration was done according to Ismail et al. [35]:

(4) $$\begin{array}{c} \beta -\text{Carotene}\left(\text{mg}/g\right)=\frac{\left[0.216\times A_{663}-0.304\times A_{450}+0.452\times A_{453}\right]\times V}{W}. \end{array}$$

Fourier transform infrared (FTIR; Perkin Elmer Spectrum One, Waltham, MA, USA) was used according to the method reported by El-Gendy et al. [25] to characterize the extracted value-added products. The X-ray diffractometer (XRD) patterns and the average particle size of the extracted alginate and cellulose have been corroborated by high-resolution XRD (PANalytical X'PERT Pro MPD, Lelyweg, EA Almelo, the Netherlands) in conjunction with a Cu k radiation source (*ʎ* = 1.5418 Å) running at 40 kV and 40 mA and a dynamic light scattering device (DLS, Zetasizer Nano Series (HT), Nano ZS, Malvern Instruments, Malvern, UK), respectively. Their granule morphologies have been studied using a field emission scanning electron microscope embedded with smart energy dispersive X-ray spectroscopy (FESEM-EDX, model Sigma 300VP Carl Zeiss, Jena, Germany).

Furthermore, according to El-Gendy et al. [25], under nitrogen circumstances, Q600 SDT simultaneous differential scanning calorimetry-thermal gravimetric analysis (DSC–TGA) and the TA instrument Discovery DSC250 (New Castle, DE, USA) were used to investigate the thermal properties of the value-added products and to generate their DSC data, respectively. The thermograms obtained were subjected to the analysis using TA Instruments Trios software (V5.2.2.47561, New Castle, DE, USA). Then, the integrated peak area was divided by the heating temperature rate (5°C/min) and the weight of the value-added product slurry (5 mg) in order to calculate the gelatinization enthalpy (ΔH J/g). For reproducibility, all analyses were done in triplicate.

### 2.5. Isolation of Microbial Strains Capable of Hydrolyzing and Saccharifying Different Algal Polysaccharides and Fermenting Different Di- and Monosaccharides

The microbial isolation has been performed from the freshly collected *S. latifolium* biomass and its associated water according to the method reported by Abo-State et al. [36]. Luria-Bertani (LB) medium with pH7 [37], Wickerham's (WH) medium with pH5.5 [38], and Sabouraud dextrose (SD) medium with pH5.5 [39] have been used for the isolation and maintenance of bacteria, yeast, and fungi, respectively. In 250 mL conical flasks, 90 mL of sterile saline (8.5 g/L NaCl) was mixed with 10 g of fresh algal biomass under aseptic conditions. For sixty minutes, the flasks were shaken at 200 rpm. Subsequently, the suspensions were cultured on LB, WH, and SD agar plates for bacteria, yeast, and fungi, respectively, and serially diluted to 10^−1^.

Inoculated LB and WH Plates were cultured for 48 hours, while the inoculated SD plates were cultured for seven days at 30°C. Then, the obtained well defined and separated colonies were streaked on a sterile, appropriate medium for purification. Fungal purification has been performed by adding 100 mg/L Rose Bengal to the Sabouraud medium before sterilization.

The primary screening and evaluation of the performance of the obtained fungal isolates on different algal polysaccharides have been performed according to Bahkali [40]. The fungal isolate discs were incubated for 7 days at 30°C onto minimal-agar plates supplemented with 1% (w/v) of different polysaccharides, alginate, cellulose, and hemicellulose. Then, the growth was monitored and tabulated.

The screening and evaluation of the performance of the obtained fungal isolates to hydrolyze and saccharify the collected *S. latifolium* biomass have been performed according to Abo-State et al. [36]. The fungal isolates were inoculated in a final concentration (5% w : w) onto sterilized wet algal biomass (25% w/v biomass : distilled H_2_O), and then statically incubated for 7 days at 30°C. The produced sugars were then extracted by 50 mL distilled H_2_O and quantified by the 3,5-dinitrosalicylic acid (DNS) method [41]. The total reducing sugar (TRS) yield (mg TRS/g algal biomass) and saccharification efficiency were then calculated according to Soliman et al. [12]:

(5) $$\begin{array}{c} \text{Yield}\left(\text{mg}/g\right)=\frac{\text{TRS mg}}{\text{Algal biomass dry weight }g}, \end{array}$$

(6) $$\begin{array}{c} \text{Sacrification }\%=\left(\frac{\text{Released TRS }g\times 0.9}{\left(\text{Algal cellulose}+\text{Algal hemicellulose}\right)\;g\;}\right)\times 100. \end{array}$$

The conversion percentage was calculated according to Kooren et al. [18]:

(7) $$\begin{array}{c} \text{Conversion }\%=\left(\frac{\text{TRS }g}{\text{Algal biomass dry weight }g}\right)\times 100. \end{array}$$

The screening of the capability of different microbial isolates to ferment wide varieties of di- and monosaccharides has been performed according to Abo-State et al. [36]. All the yeast and bacterial isolates were streaked separately onto minimal-agar plates [40] supplemented with 1% (w/v) of different saccharides, and then incubated for 48 h at 30°C. Finally, the growth was monitored and tabulated. The sugars used for this test were mannitol as representative for sugar alcohol; xylose and arabinose as representative for reducing pentoses; glucose, mannose, galactose, and fructose as representative for reducing hexoses; lactose and maltose as representative for reducing disaccharides; and sucrose as representative for nonreducing disaccharides.

### 2.6. Identification of the Obtained Microbial Isolates

The bacterial isolates were molecularly identified by 16S ribosomal RNA gene sequence using two sets of universal primers, 27F (AGAGTTTGATCMTGGCTCAG) and 1492R (TACGGYTACCTTGTTACGACTT), amplifying about 1465 bp of the 16S rDNA region [42]. For the molecular identification of the isolated yeast and fungi, the internal transcribed spacer regions in 18S ribosomal RNA, ITS1 (CTTGGTCATTTAGAGGAAGTAA) and ITS4 (TCCTCCGCTTATTGATATGC), were used to amplify 400–600 bp containing the complete sequence of the 5.8S rRNA gene and ITS 2 [43, 44]. Genomic DNA of bacteria and yeast was extracted using the GeneJET Genomic DNA Purification Kit (Thermo Fisher Scientific, USA) according to the manufacturer's instructions, whereas DNA of fungi was extracted by the CTAB method [45]. PCR was performed by adding 40 ng of extracted DNA in 50 mL of the PCR reaction solution (1 U of DreamTaq DNA polymerase (Thermo Fisher Scientific, USA), 1x buffer, 0.2 mM dNTPs, and 10 pmol of the appropriate primers). PCR products were purified by the GeneJET Gel Extraction Kit (Thermo Fisher Scientific, USA), and the purified DNA has been sent to GATC Biotech Company (Ebersberg, Germany) for sequencing. The selected efficient isolates were then identified based on their obtained 16S rDNA and ITS region sequence (query sequence) analysis on the NCBI server https://www.ncbi.nlm.nih.org, for bacteria, yeast, and fungi, respectively, via the BLAST tool of the NCBI https://blast.ncbi.nlm.nih.gov/blast.cgi. The phylogenetic tree was then constructed using the maximum likelihood (ML) algorithms by MEGA 11.0.13 software after muscle alignment with 500 bootstrapping trials.

### 2.7. Identification and Quantification of the Produced Fermentable Sugars

Identification of sugar profile and their quantification in the produced hydolyzates have been performed as previously described by Abo-State et al. [36], using the high performance liquid chromatography (HPLC) model Agilent 1200 series USA equipped with a refractive index RI detector model (Agilent 1260 infinity, Santa Clara, CA, USA) and a SUPELCOSIL-LC-NH_2_ (25 cm × 4.6) column.

### 2.8. Screening the Capability of the Obtained Microbial Isolates for Bioethanol Production

The screening was performed according to the method reported by Abo-State et al. [36] on a mixture of representative model sugars (glucose, xylose, and mannitol) for hexoses, xyloses, and alcoholic sugars, respectively, besides the produced algal hydrolyzate. After adding peptone (10.0 g/L), KH_2_PO_4_ (2.0 g/L), and MgSO_4_.7 H2O (1.0 g/L) to the model sugary solution or hydrolyzate, the mixture was autoclaved for 20 minutes at 121°C to sterilize it. 10% (v/v) of the microbial isolates were added to the medium as an inoculant. The inoculated cultures were then incubated for 48 hours at 150 rpm at 30°C. Finally, the fermented medium was centrifuged for 10 minutes at 10,000 rpm. The residual TRS were quantified by the DNS method [41] and the sugars' profile before and after fermentation was determined by the HPLC analysis according to the method reported by Abo-State et al. [36]. The produced ethanol was quantified according to the method described by Soliman et al. [12] using gas chromatography (model 6890; Agilent), equipped with a flame ionization detector and a nominal capillary column (60 m × 530 *µ*m × 5.00 *µ*m). Noninoculated flasks were used as −ve control and subjected to all experimental conditions as inoculated ones.

Bioethanol yield was calculated according to Zeng et al. [46]:

(8) $$\begin{array}{c} \text{Bioethanol yield}=\frac{\text{Concentration of produced bioethanol }\left(g/L\right)}{\text{Algal biomass }\left(g/L\right)}. \end{array}$$

Actual bioethanol yield was calculated according to Jeyakumar et al. [47]:

(9) $$\begin{array}{c} \text{Actual biothanol yield}=\frac{\text{Concentration of produced bioethanol }\left(\text{mg}/L\right)}{\text{Initial Concentration of sugars }\left(\text{mg}/L\right)}. \end{array}$$

Bioethanol conversion yield was calculated according to Aparicio et al. [48]:

(10) $$\begin{array}{c} \text{Biothanol conversion yield }\%=\left(\frac{\text{Concentration of produced bioethanol }\left(\text{mg}/L\right)}{\text{Initial Concentration of sugars }\left(\text{mg}/L\right)}\right)\times 100. \end{array}$$

### 2.9. The Macroalgal Synthesis of Carbon-Dot

To maximize the value of the process, the spent waste *S. latifolium* disposed of from the bioethanol production process was used in a groundbreaking step to hydrothermally prepare the carbon-dot (CD) according to the method reported by Kumar et al. [49]. In a 150 mL Teflon-lined stainless steel autoclave, 10 g of the spent waste *S. latifolium* were combined with 100 mL of distilled water. The reaction mixture was heated at 180°C for 4 hours at a steady temperature until a dark brownish solution was produced. The reaction mixture was cooled to ambient temperature. To remove large unreacted particles, the obtained yellowish-brown solution was filtered through a Whatman 42 filter paper. To solubilize the produced CD and improve fluorescence, the solution was centrifuged at 5000 rpm for 20 minutes before being suspended in 5 ml of 1 N NaOH. Then, the aforementioned solution was dialyzed against pure water for 24 hours to purify the obtained CD.

The photoluminescence characteristics of the produced CD were determined at room temperature using a spectrofluorometer (JASCO FP-6500, Jasco International Co., Ltd., Tokyo, Japan) with an excitation wavelength set at *λ* = 254 nm. The produced CD was also characterized by the SENTERRA II Dispersive Raman Microscope (Bruker Optics, Billerica, MA, USA), Fourier transform infrared (FTIR; Perkin Elmer Spectrum One, Shelton, CT, USA), field emission scanning electron microscope-embedded smart energy dispersive X-ray spectroscopy (FESEM-EDX, model Sigma 300VP Carl Zeiss, Jena, Germany), and high-resolution transmission electron microscope (HRTEM, JEOL-JEM 2100F, 80–200 kV, Tokyo, Japan). The biocidal activity of the produced CD against pathogenic microorganisms was evaluated according to El-Gendy et al. [25].

The activity index was calculated according to El-Gendy et al. [25]:

(11) $$\begin{array}{c} \text{Activity index}=\frac{\text{CD inhibition zone }\left(\text{mm}\right)}{\text{Standard antibiotic inhibition zone }\left(\text{mm}\right)}. \end{array}$$

### 2.10. Statistical Analysis

A Tukey-test, using SPSS software version 13.0 (Informer Technologies, Inc., Los Angeles, CA, USA) was performed to determine the significant difference between the acquired data, with a *p* value of greater than 0.05 considered nonsignificant at the 95% confidence level.

## 3. Results and Discussion

### 3.1. Morphological Analysis of the Collected Brown Macroalgae

**Figure 3:**
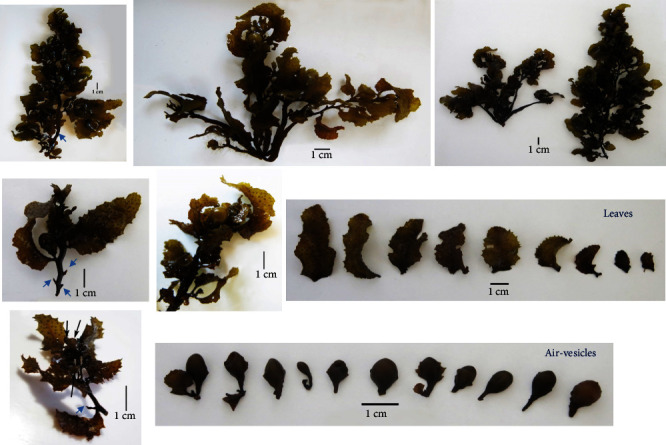
Normal visual examination of the collected brown *Sargassum latifolium*.

**Figure 4:**
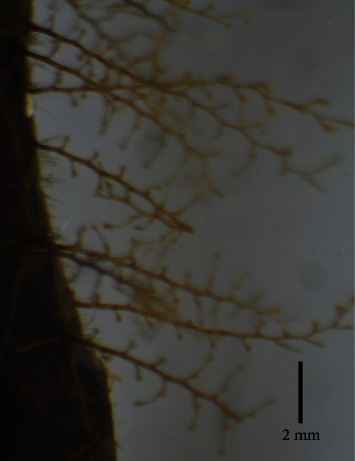
Stereo zoom microscopic examinations showing the paraphyses on the axis of the collected brown *Sargassum latifolium*.

**Figure 5:**
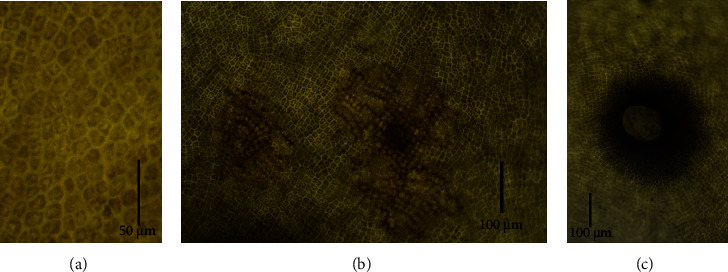
Microscopical examination for different transverse sections of the collected brown *Sargassum latifolium*: (a) medullar cells, (b) female conceptacles, and (c) male conceptacle.

The visual observation of the collected brown macroalgae (Figure 3) matches well with the previously published marine brown macroalgal species *Sargassum latifolium* collected from the Red Sea at the Kingdom of Saudi Arabia (KSA) Umluj shore [50], KSA Jeddah coast [2], Egypt Hurghada shoreline [51, 52], Egypt Ras Ghareb coast [12], and Egypt Ras Sedr coast [3]. *Sargassum latifolium* belongs to the *Phylum Ochrophyta, Class Phaeophyceae, Order Fucales*, and *Family Sargassaceae*. The collected thallus was found to reach up to 70 cm in height. It has spherical, obovate, and pyriform air-vesicles that are stalked with serrated wings and without any projections (Figure 3). It has spiny axes (blue arrows, Figure 3). It has lanceolate leaves with seriated edges, a leathery texture, and randomly distributed cryptostomata (Figure 3). The stereomicroscopy examination of the axis (Figure 4) shows the paraphyses or mucilage hairs, which are known to be responsible for mucilage secretion and also the absorption of minerals and water. The microscopic examination of the transverse sections of *Sargassum latifolium* blades or leaves (Figure 5(a)) with their female conceptacle (Figure 5(b)) and male conceptacle (Figure 5(c)) is also illustrated and reveals the presence of oogonia and antheridia, which are responsible for sexual reproduction.

### 3.2. The Physicochemical Analysis of the Associated Water for the Collected Brown *S. latifolium*

The data illustrated in Table 1 summarize the physicochemical analysis of the algal biomass collected water and are comparable to those reported for Egyptian Red Sea water associated with *S. latifolium* [3] and *S. aquifolium* [1]. The observed high metal content might be related to the released metals from sediments, anthropogenic sources due to coastal region human activities, disposed industrial effluents, and other activities related to the oil and gas industry in that area [12]. The recorded pH and salinity matched those reported for the Red Sea by Abu Ahmed et al. [3]. The recorded oil content of approximately 11.85 mg/L might indicate oil pollution.

According to biological response values that may accompany or follow oil pollution, lethal effects range from 1 to 10 mg/L, but sublethal effects can be as low as 1 mg/L [53]. The recorded BOD of 48.4 mg/L (Table 1) might indicate organic pollution. Healthy water streams are characterized by a BOD <2 mg/L, while polluted ones have a BOD ≥10 mg/L [54]. The relatively high concentrations of nitrite and nitrate (Table 1) would enhance the growth and proliferation of macroalgae [55]. The low concentrations of ammonium and phosphate (Table 1) would confirm the overgrowth of macroalgae, as they are considered the major nutrients consumed during their primary growth and proliferation [54].

### 3.3. The Physicochemical Analysis of the Collected Brown *S*. *latifolium*

The tabulated proximate analysis of S. *latifolium* biomass (Table 2) depicts a sufficient calorific value of 14.82 ± 0.5 MJ/kg. That is as good as those reported for *Sargassum* [56] and lignocellulosic [57] biomass. Furthermore, the undesirable ash (Table 2) and heavy metal (Table 3) contents were low and within the permissible levels recommended by the International Organization for Standardization [58]. Thus, it can be utilized as a primary solid biofuel.

The recorded cellulose, hemicellulose, and lignin contents (Table 2) for the collected *S. latifolium* biomass in this study are comparable to the previously reported data for *Sargassum* spp. [50, 60], which recorded 20.35 ± 0.5% and 15.41 ± 0.5% for cellulose, 25.73 ± 0.5% and 25.43 ± 0.5% for hemicellulose, and 6.95 ± 0.5% for lignin. The holocellulose content of approximately 40.5 ± 0.5% aligns with the reported range of 40% to 60% for *Sargassum* species [61].

The nutritional analysis illustrated in Table 2 shows that the collected brown *S. latifolium* biomass has notable carbohydrate, ash, protein, and fiber contents with a rational amount of lipid, recording 41.5 ± 0.5%, 24 ± 0.5%, 15.94 ± 0.31%, 7.98 ± 0.5%, and 1.38 ± 0.05% (w/w), respectively. These values were comparable to those reported for *Sargassum* sp. [65]. Moreover, it has a considerable amount of beneficial macronutrients and micronutrients, recording 508.46 ± 0.2 mg/kg and 44.95 ± 0.2 mg/kg, respectively, with a low concentration of undesirable heavy metals, recording 989.9 ± 0.25 *µ*g/kg (Table 3). That suggests using it not only medicinally as a multivitamin source and in food supplements [62], but also in animal fodder production [63, 64] and food manufacturing [65].

The recorded concentrations of undesirable heavy metals, Pd, Cd, and Hg (Table 3), were comparable to those reported by Lee et al. [10] for different collected *Sargassum* species. The carbohydrate and ash contents of the collected *S. latifolium* in this study (Table 2) were comparable to those reported by Fouda et al. [52] and Abu Ahmed et al. [3]. However, the recorded protein and fiber contents (Table 2) were higher. But they were comparable to those reported for *S. binderi*, which is recommendable for poultry diets [66]. Moreover, the carbohydrate and lipid contents (Table 2) were comparable to those of Egyptian *S. latifolium* [67], which recorded 41.08% and 1.13%, respectively.

The data in Tables 3 and 4 closely match the Egyptian organic fertilizer standard, with N + P_2_O_5_ + K_2_O and organic C contents of 6.00 ± 0.07% and 29.61 ± 0.05%, respectively, suggesting its suitability for organic fertilizer production. Different *Sargassum* species *S. vulgare* [68, 69], *S. polycystum* [70], *S. horneri* [71], and *Sargassum* sp. [72] have been reported for the production of organic fertilizer.

The recorded relatively high carbohydrates, cellulose, and hemicellulose contents, with a relatively low lignin content of approximately 41.5 ± 0.5%, 15.5 ± 0.5%, 25 ± 0.5%, and 8.2 ± 0.5%, respectively (Tables 2), recommend the applicability of the collected *S. latifolium* biomass in the production of liquid biofuels and different biorefineries [12, 25, 73], besides the food, cosmetics, and pharmaceutical industries [56].

### 3.4. A Fully Integrated Process for Extraction of Various Value-Added Products from *S. latifolium* Biomass

#### 3.4.1. The Pigments and Antioxidants

**Figure 6:**
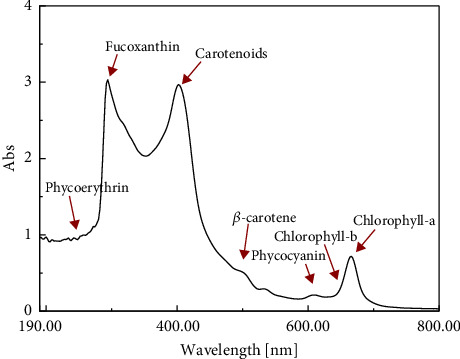
UV/Vis spectrum of *S. latifolium* ethanol extract.

The ethanol extraction yielded pigments and antioxidants mixture of approximately 6.74 ± 0.5% (w/w). The UV/Vis spectrum of the obtained extract (Figure 6) matched that reported by Indrawati et al. [74] for *Sargassum* sp. pigment extract. It proved the presence of fucoxanthin antioxidant in the ethanol extract of *S. latifolium* macroalgae, besides some other important pigments and antioxidants: chlorophyll-a and -b, *β*-carotene, phycoerythrin, and phycocyanin. These findings are comparable to those reported for *S. binderi* [75]. The characteristic peaks of chlorophyll-a and -b appear at ʎ_662nm_ and ʎ_645nm_ [76, 77], with the predominance of chlorophyll-a. That coincides with the extract of brown macroalgae, *Saccharina latissima* [77]. The carotenoids with their predominant peak and *β-*carotene appear in the blue range between ʎ_400nm_ and ʎ_500nm_ [76]. The characteristic peaks of different valuable antioxidants also appear: fucoxanthin at ʎ_330nm_ and ʎ_420nm_ [76, 77], phycoerythrin at ʎ_270nm_, ʎ_308nm_, ʎ_497nm_ [78], and ʎ_540nm,_ while phycocyanin at ʎ_618nm_ [78], with predominance of fucoxanthin. Sanger et al. [79] reported the presence of chlorophyll (a, b, and C1 + C2), fucoxanthin, carotenoids, phycocyanin, and phycoerythrin in *Sargassum* species.

The chlorophyll and carotenoids recorded 77.6 ± 0.5 mg/g and 180 ± 0.5 mg/g, respectively. The fucoxanthin and *β*-carotene concentrations were found to be 5.86 ± 0.5 mg/g and 0.93 ± 0.5 mg/g, respectively.

The predominance of chlorophyll-a and fucoxanthin in the extract of *S. latifolium* has been previously reported by Ismail et al. [35]. The predominance of fucoxanthin over phycoerythrin and phycocyanin has also been reported for the ethanol extract of *Sargassum* sp. [79]. Fucoxanthin is considered one of the major carotenoids in *Sargassum* species [80, 81]. It is known to be stable in normal storage conditions, and it has health-enhancing properties. It has many applications in the pharmaceutical and cosmetic industries, and it can also be applied as a bioingredient and functional food [81, 82].

#### 3.4.2. Alginate Yield and Characterization

The collected *S. latifolium* yielded a pale yellow-colored alginate of approximately 21.97 ± 0.5% (w/w). This is higher than that previously published for *S. latifolium* [67, 83], which recorded 17.5%. However, *S. filipendula* [84], *Sargassum* sp. [85], and *S. muticum* [86] reported similar values of 24%, 24.56%, and 24.66%, respectively. According to Kok and Wong [87], the yield of alginate differs according to the season of algal harvesting, algal species, and method of extraction. *S. angustifolium* alginate ranged between 22.8% and 24.4% within the winter and summer, respectively [88]. According to Dharmayanti et al. [89], the alginate yield depends on the habitat itself, the sea tides, light intensity, and the available aquatic nutrients.

**Figure 7:**
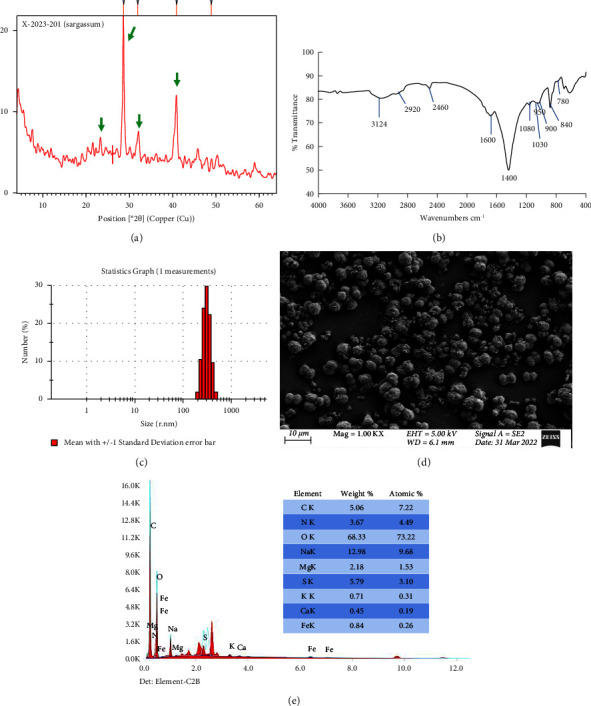
The XRD patterns (a), FTIR spectrum (b), particle size distribution (c), FESEM picture (d), and EDX analysis (e) of the extracted alginate.

The XRD pattern of the alginate extracted from the collected *S. latifolium* (Figure 7(a)) is similar to that published by Benfares et al. [90]. It would indicate, according to Dalal et al. [67], the semicrystalline nature of the extracted alginate. According to Aprilliza [91], brown algae alginate has a semicrystalline structure. Such crystallinity in the extracted alginate might be, according to Kavitha et al. [92], attributed to the robust intermolecular interactions within the alginate chains via hydrogen bonding, which, according to Fang et al. [93], would differ depending on the algal species. The diffraction peaks appear around 2ϴ of 26°, 30°, 32.5°, and 37° (Figure 7(a), green arrows) and are related to the 110 and 200 reflection planes of polyguluronate and polymannuronate units, respectively [94]. Other peaks that appeared around 45.5° and 52.5° also appeared in the XRD patterns of different seaweed alginates [94] and are related, according to Sundarrajan et al. [95], to the presence of other amorphous halos.

The FTIR analysis (Figure 7(b)) showed the characteristic peaks of alginate, which appear between 1080 cm^−1^ and 840 cm^−1^. The FTIR spectrum (Figure 7(b)) is also matched with alginate extracted from *S. duplicatum*, *S. crassifolium*, and *S. pilulifera* [96, 97]. The symmetric stretching vibration of carboxylate groups is obvious at 1400 cm^−1^ with the presence of the asymmetric stretching vibration O-C-O at 1600 cm^−1^. The vibration peaks around 1080 cm^−1^ and 1030 cm^−1^ would indicate the presence of guluronic and mannuronic units, respectively [6]. That is also confirmed by the presence of the peak of uronic acid C-O stretching vibration around 950 cm^−1^ and another small shoulder peak around 780 cm^−1^. In addition, the vibrational peak of *β*-mannuronic acid residues appears around 820–880 cm^−1^ [98]. The peak that appears around 900 cm^−1^ is related to the C–H deformation vibration of *α*-L guluronic acid. Moreover, the broad hydrogen-bonded O–H stretching vibration and C–H stretching vibration appear at 3000–3200 cm^−1^ and 2920 cm^−1^, respectively [6]. While the peak around 2460 cm^−1^ might be related to C-H of proteins [99]. The absence of peaks around 1200 cm^−1^ and 600 cm^−1^, which are related to the symmetric and asymmetric O=S=O in sulfated polysaccharides [100], might indicate the purity of the extracted alginate and the absence of laminarin or fucoidan [1]. The D-mannuronate to L-guluronate ratio (M/G) can be calculated from the FTIR spectrum [101], and it has been found to be 1.01. That was comparable to *S. asperifolium* [83] and *S. latifolium* [102] alginates M/G values of 1.05. The *Sargassum* alginate M/G ratio is reported to range between 0.8 and 1.5 [103]. It has been reported that alginate with a large content of guluronic acid and a low M/G (<1) produces a strong and rigid gel [104], but elastic and soft gel are formed with alginate with a high M/G (>1) and a lower amount of guluronic acid blocks [105].

The DLS analysis (Figure 7(c)) revealed alginate with an average particle size of 311 nm. The FESEM image (Figure 7(d)) reveals rough, irregular, and spherical-shaped alginate particles with different sizes. The outer surface of alginate particles seems to have spines and perforations. A similar observation was reported for alginate extracted from *S. duplicatum* and *S. crassifolium* [96]. The EDX analysis revealed a protein content of approximately 22.94% (w/w) in the alginate extracted from *S. latifolium*. The EDX also depicted the presence of minerals: Na, Mg, Fe, Ca, and K of approximately 12.98, 2.18, 0.84, 0.45, and 0.17% (w/w), respectively, in addition to S of approximately 5.79% (w/w). According to Mohamed et al. [86], the protein and mineral contents of alginate differ with the applied extraction methods.

**Figure 8:**
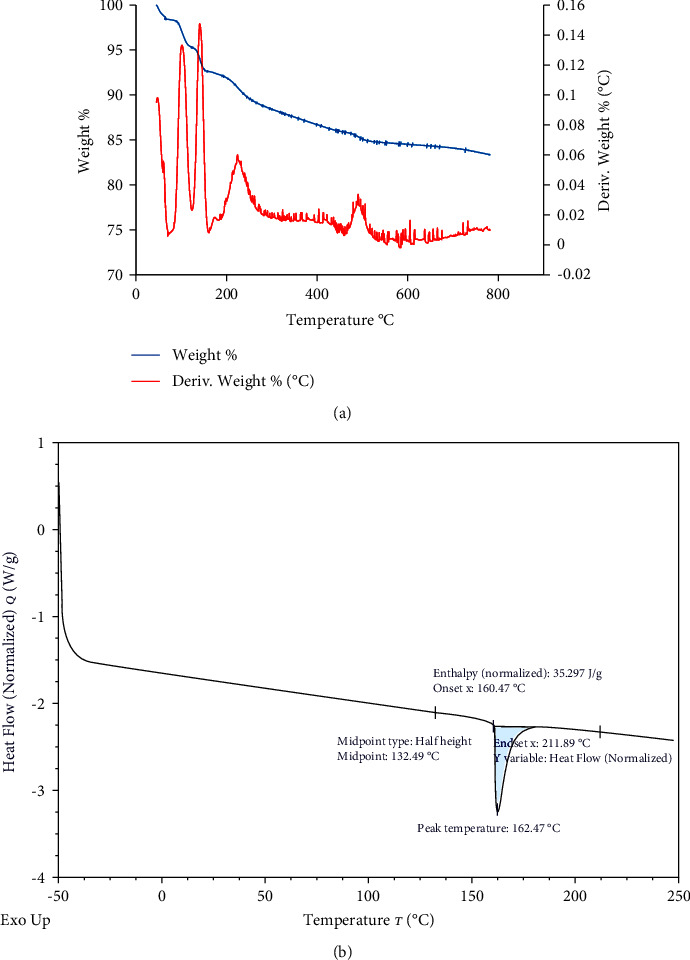
TGA/DTG (a) and DSC (b) analyses of the extracted alginate.

The TGA/DTG plot (Figure 8(a)) matched well with that previously reported for Egyptian *S. latifolium* alginate [67]. It revealed the main thermal degradation before 100°C, which might be attributed to water evaporation [6]. The other peak, which appears at 140°C, is also reported by Nesic et al. [6], Siriwardane et al. [106], and Russo et al. [107] and was attributed to the release of bounded water, carbonate dehydration, and decomposition of protein and polyphenolic compounds, respectively. Another main thermal degradation occurred around 220°C, which, according to Nesic et al. [108], might be related to the breakdown of alginate molecules, the mannuronic blocks, followed by the guluronic blocks. The peak that appeared around 480°C might be related to the decomposition of the carbonized residue produced during the two previous main degradation stages [67].

The DSC curve (Figure 8(b)) revealed the main peak at 162.47°C, which would correspond to the melting point of alginate [109]. Xu et al. [110] reported the thermal stability of alginate up to 160°C.

#### 3.4.3. Cellulose Yield and Characterization

The collected *S. latifolium* yielded approximately 16.40 ± 0.5% cellulose (w/w). The extracted cellulose has a purity of approximately 79.95 ± 0.05%, with hemicellulose and lignin contents of ≈0.36 ± 0.05% and 0.06 ± 0.02%, respectively.

**Figure 9:**
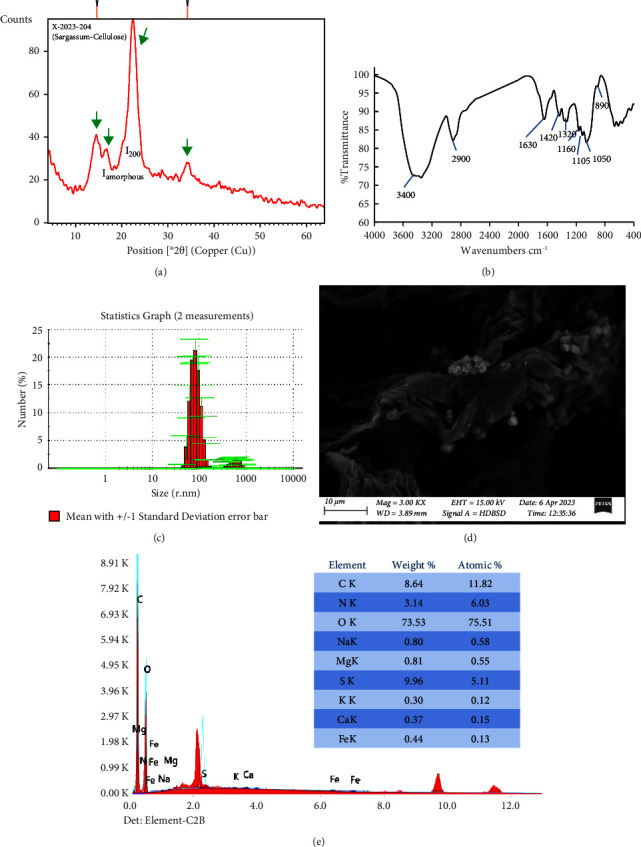
The XRD patterns (a), FTIR spectrum (b), particle size distribution (c), FESEM picture (d), and EDX analysis (e) of the extracted cellulose.

The XRD pattern of the extracted cellulose (Figure 9(a)) was found to be well matched with that of cellulose (card number 00-003-0192) and chemical formula (C_6_H_10_O_5_). The extracted cellulose displays the distinguished peaks of cellulose I allomorph at (2*θ*) 15.14°, 16.25°, 22.68°, and 34.84° (Figure 9(a), green arrows), which correspond to crystallographic planes of 1̅10, 110, 200, and 004. This is similar to the cellulose extracted from *S. fluitans* [111] and *S. natans* [112].

The crystallinity index (CrI%) was calculated according to El-Gendy et al. [25]:

(12) $$\begin{array}{c} \text{CrI }\%=\left(\frac{I_{200}-I_{\text{amorphous}}}{I_{200}}\right)\times 100, \end{array}$$

where the peak intensity at (2*θ*) 22.68° represents I_200_, while the peak intensity at (2*θ*) around 18° represents the *I*_amorphous_ (Figure 9(a)). That revealed a CrI% of 93.55 ± 0.05%, confirming the high purity of the extracted crystalline cellulose [113]. That was comparable with the reported CrI% of cellulose extracted from brown *Saccharina japonica* [114] and *Sargassum vulgare* [115], which recorded 92.34% and 91.37%, respectively.

The FTIR spectrum (Figure 9(b)) shows the distinctive peaks of cellulosic polysaccharides extracted from seaweed, at 890, 1105, and 1160 cm^−1^ [113]. It matched well with the previously reported FTIR spectrum of *S. fluitans* cellulose nanocrystals [111] and *S. wightii* cellulose [61]. The peaks around 3400 and 1630 cm^−1^ are denoted for O-H stretching and bending vibrations, respectively, while the peak around 2900 cm^−1^ is consistent with the CH_2_ stretching vibration. The peak appears around 1050 cm^−1^ is related to the symmetric stretching vibrations of C–O–C linkages. The peaks between 1420 cm^−1^ and 1320 cm^−1^ are related to the H–C–H scissor and H–C–H tip vibrations. The characteristic small FTIR-vibration peak of the *β*-glycosidic bond which is an indicative for the presence of the cellulose polymeric polysaccharides appears around 890 cm^−1^. The absence of N-H bending vibration of lipids between 1500 cm^−1^ and 1550 cm^−1^ confirm that the extracted cellulose is free of lipid. Also, the absence of the peak around 1710 cm^−1^ of C=O indicates that the bleaching process using the hydrogen peroxide did affect the extracted cellulose and did not oxidize it or the xyloglucan [25].

The DLS analysis of the extracted cellulose (Figure 9(c)) showed a bimodal particle size distribution at 87.18 nm and 639.6 nm, with a major average particle size in the nanorange of approximately 87 nm.

The FESEM image (Figure 9(d)) revealed cellulosic fibrils. That resembles the one reported for *S. wightii* cellulose [116]. The EDX analysis (Figure 9(e)) revealed the high purity of the extracted cellulose, as it is mainly composed of carbon and oxygen with traces of minerals, and its protein content reached approximately 19.63%.

**Figure 10:**
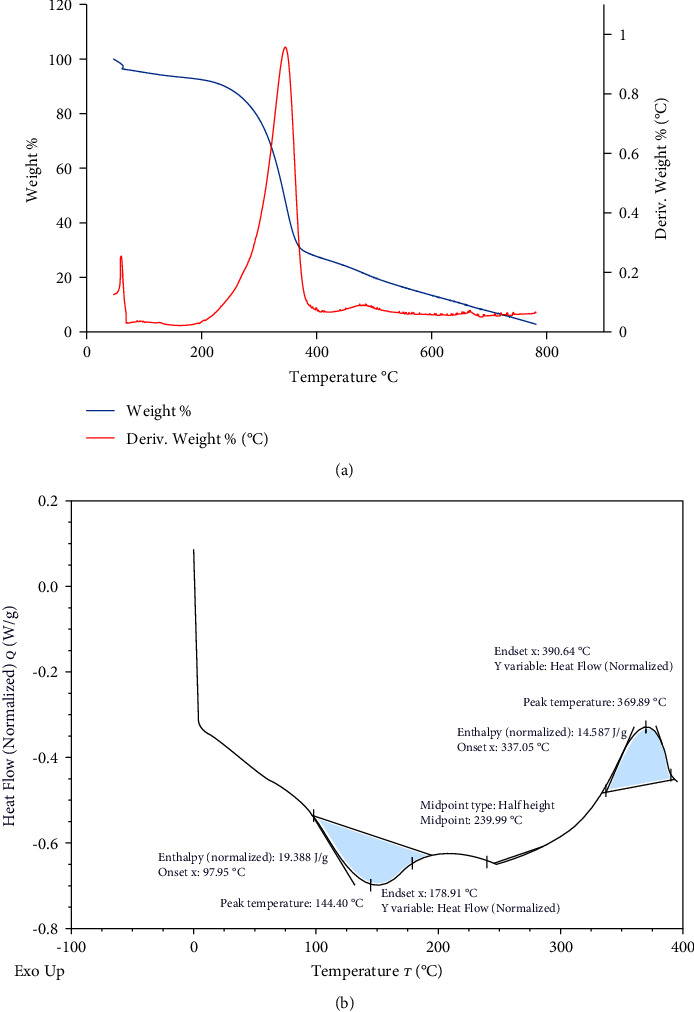
TGA/DTG (a) and DSC (b) analyses of the extracted cellulose.

The main distinguished peak that appears in the TGA/DTG spectrum around 340°C (Figure 10(a)) might be attributed to cellulose degradation [113]. The weak peak that appears around 470°C might be related to the degradation of other secondary complexed cellulosic products, for example, cellulose esters [113]. The DSC analysis (Figure 10(b)) proved the thermal stability of the extracted cellulose, where complete decomposition would occur at ≈ 369.89°C. This increases its usefulness as a strengthening ingredient in manufacturing packaging materials and biocomposites [25].

### 3.5. Isolation of Fungal Strains Capable of Hydrolyzing and Saccharifying Different Algal Polysaccharides

**Figure 11:**
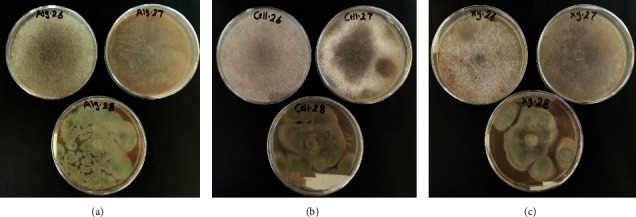
Growth of fungal isolates on different algal polysaccharides: (a) alginate plates, (b) cellulose plates, and (c) Xylan plates.

The microbial isolation has been performed on the freshly collected *S. latifolium* biomass and its associated water. Three fungi, a yeast, and a bacterium were isolated. The three fungal isolates showed wide versatility for the utilization of algal polysaccharides (Figure 11 and Table 5), with sufficient hydrolyzing and saccharifying capabilities for the collected *S. latifolium* biomass (Table 5). The saccharification percentage ranged between 28.8 ± 0.5% and 38.52 ± 0.5%, with a corresponding TRS of approximately 1.6 ± 0. 5 g/L and 2.1 ± 0.5 g/L (Table 5). The noninoculated flasks proved the production of approximately 100 mg/L TRS. Thus, the physical hydrothermal treatment of *S. latifolium* biomass led to a saccharification % of 4.44 ± 0.09%. There was a high statistical significant difference (*p* < 0.0001) in the yielded TRS, saccharification%, and conversion% between inoculated and noninoculated flasks. That proves the efficient capabilities of the obtained fungal isolates to hydrolyze and saccharify the algal biomass. Moreover, there was a high statistical significant difference (*p* < 0.0001) in the yielded TRS, saccharification%, and conversion% performed by *P. oxalicum* SBWF3 relative to those *A. galapagensis* SBWF1 and *M. hiemalis* SBWF2. However, there is a relatively statistical significant difference (*p* < 0.0069) between the performance of SBWF1 and BWF2.

**Figure 12:**
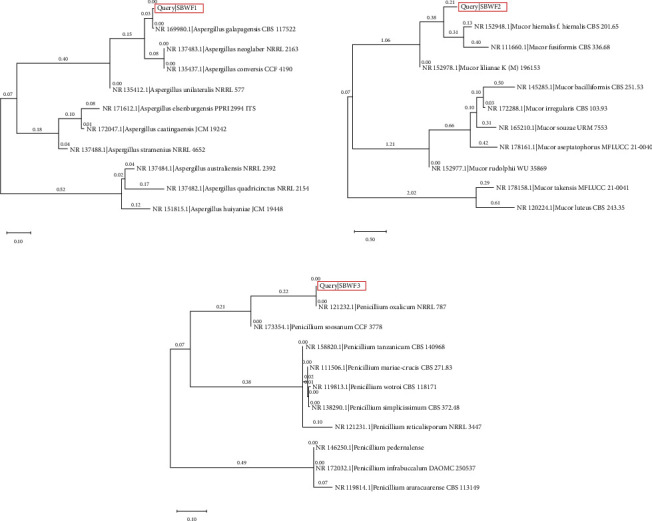
Maximum likelihood phylogenetic trees for ITS regions of three fungal isolates.

The obtained fungal isolates were identified as *Aspergillus galapagensis* strain SBWF1 (GenBank accession number OR636487), *Mucor hiemalis* strain SBWF2 (GenBank accession number OR636488), and *Penicillium oxalicum* strain SBWF3 (GenBank accession number OR636489). Their phylogenetic trees are illustrated in Figure 12.

Typically, costly enzymes are used to complete the hydrolysis or saccharification of acid-hydrolyzed macroalgae [47, 59, 117]. However, the produced TRS yield in this study using the eco-friendly hydrothermal pretreatment and the intact fungus as an enzymatic source showed to be very promising and ranged between approximately 317.48 ± 1.75 and 428.62 ± 1.5 mg TRS/g *S. latifolium* biomass (Table 5). That was higher than those reported by Soliman et al. [12], where *A. niger* HN3 and *Penicillium* sp. HN1 yielded 136.4 and 109 mg TRS/g *S. latifolium* biomass, respectively. Moreover, the recorded conversion% 31.75 ± 0.2%–42.86 ± 0.2% (Table 5) were much higher than those reported by Kooren et al. [13] applying different costly physical and chemical saccharification processes to *S. wightii* biomass, which ranged between 9.33% and 18.67%. Thus, the obtained fungal strains overcome the presence of alginate, which is one of the main obstacles to bioethanol production [118].

### 3.6. Isolation of Microbial Isolates Capable of Fermenting Different Di- and Monosaccharides

**Figure 13:**
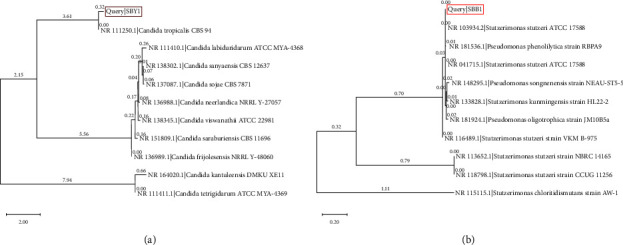
Maximum likelihood phylogenetic tree for the ITS regions of yeast isolate SBY1 (a) and for the 16S rDNA region of isolate SBB1 (b).

There was no detected bioethanol production or sugar decrease in noninoculated flasks (−ve control). According to the data listed in Tables 6 and 7, the isolated bacteria and yeast showed good capabilities for fermenting pentoses, hexoses, and mannitol into bioethanol. They were identified as *Stutzerimonas stutzeri* strain SBB1 (GenBank accession number OR764547) and *Candida tropicalis* strain SBY1 (GenBank accession number OR636490), respectively. Their phylogenetic trees are illustrated in Figure 13. *C. tropicalis* KCTC7212 has been previously reported for fermenting mannitol into bioethanol [119]. *C. tropicalis* has been previously isolated from the Egyptian Red Sea for the utilization of seaweed for bioethanol production [120]. The marine bacterium *Pseudomonas stutzeri*, which is now known as *Stutzerimonas stutzeri* [121], has been isolated for bioethanol production from seaweed [11, 122].

It can be depicted from data listed in Table 7, that there was a high statistical significant difference (*p* < 0.0001) in the bioethanol fermentation capabilities of *S. stutzeri* SBB1 and *C. tropicalis* SBY1, yielding 0.73 ± 0.029 and 0.39 ± 0.014 g bioethanol/g algae, respectively. Moreover, SBB1 showed higher capabilities for utilization of mannitol and xylose than SBY1 (*p* < 0.0001), with nearly the same capabilities of utilizing glucose (*p* > 0.05). Thus, *S. stutzeri* SBB1 is recommendable to overcome the second main obstacle of bioethanol production from brown macroalgae, and it has further been tested for fermenting the obtained algal hydrolyzates. That produced a sufficient bioethanol yield of approximately 0.25 ± 0.014, 0.26 ± 0.018, and 0.37 ± 0.020 g ethanol/g algal biomass, with a corresponding actual bioethanol yield of 0.79 ± 0.007, 0.77 ± 0.007, and 0.86 ± 0.007 g ethanol/g TRS, from the hydolyzates obtained from SSF using *A. galapagensis* SBWF1, M. *hiemalis* SBWF2, and *P. oxalicum* SBWF3, respectively (Table 7). It is worth mentioning that there was no statistical significant different between the bioethanol yields obtained from the SBWF1 and SBWF2 hydrolyzates (*p* > 0.05). Yet, there is a statistical significant difference between the yielded bioethanol from SBWF3 hydrolyzate and the other aforementioned SBWF1 and SBWF2 hydrolyzates (*p*0.001). The tabulated bioethanol yields (Table 7) were higher than those reported for acid and enzymatic hydrolysis of *S. horneri*, followed by fermentation using *Pichia stipitis*, which yielded 0.11 g ethanol/g algal biomass [46]. These were also higher than the actual bioethanol yield reported by Kadimpati et al. [123] for the acid hydrolysis of *S. cinereum*, followed by fermentation using *Saccharomyces cerevisiae* MTCC170, which yielded 0.465 g ethanol/g TRS. Those were also higher than that reported by Soliman et al. [12] for hydrothermal and fungal hydrolysis and saccharification of *S. latifolium* by SSF using *Trichoderma asperellum* RM1, followed by fermentation using a consortium of two *Saccharomyces cerevisiae* strains, ATCC 76621 and RM2, which yielded 0.29 ethanol/g TRS.

The recorded bioethanol conversion yields of *A. galapagensis* SBWF1 and *M. hiemalis* SBWF2 hydrolyzates of 79 ± 0.70% and 77 ± 0.70%, respectively (Table 7), were comparable to those reported for hydrothermal and enzymatic hydrolysis of *Sargassum* species, followed by fermentation using *Saccharomyces cerevisiae* PE-2, which yielded 76.23% of bioethanol conversion [48]. Nevertheless, the recorded bioethanol conversion yield of *P. oxalicum* SBWF3 hydrolyzate of 86 ± 0.70% (Table 7) was comparable to that reported for acid and enzymatic hydrolysis of *Sargassum* species followed by fermentation using *Saccharomyces cerevisiae*, which yielded 89% of bioethanol conversion [59].

**Figure 14:**
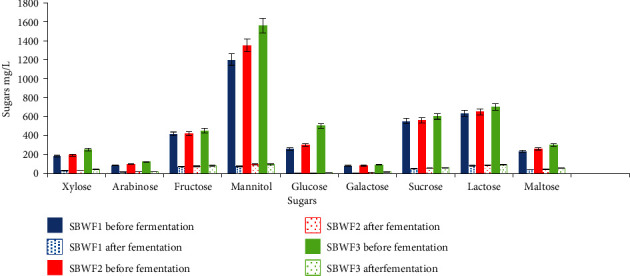
Sugar profile of the obtained *S. latifolium* hydrolyzates before and after bioethanol fermentation process.

Table 7 shows that the recorded bioethanol conversion yields exceeded the theoretical yield associated with glucose, which is 51%. That might be due to the presence of other fermentable sugars (Figure 14), which would have been fermented to ethanol after the complete consumption of glucose [124]. The sugars' profiles of the three hydrolyzates before and after the bioethanol fermentation process (Figure 14) confirmed the wide versatility of *S. stutzeri* SBB1 for utilizing reducing hexoses and pentoses, nonreducing sucrose, and mannitol.

Elshobary et al. [125] stated that multiplying the produced amount of bioethanol (g/g biomass) by the corresponding calorific value of 26.7 MJ/kg will give an estimate of the energy output based on the estimated energy stored in the bioethanol. Thus, based on the data listed in Table 7, the energy output was estimated to be approximately 6.68 ± 0.37, 6.94 ± 0.22, and 9.88 ± 0.53 MJ/kg for bioethanol yields of *A. galapagensis* SBWF1, *Mucor hiemalis* SBWF2, and *P. oxalicum* SBWF3 hydrolyzates, respectively. It is noteworthy to emphasize the high statistical significant difference (*p* < 0.0001) between the energy output for bioethanol yield of *P. oxalicum* SBWF3 hydrolyzate relative to those of SBWF1 and SBWF1, besides the nonstatistical significant difference between those of SBWF1 and SBWF1 (*p* > 0.05). Thus, the suggested solid state fungal hydrolysis and saccharification of using *P. oxalicum* SBWF3 that sequentially followed by a separate fermentation process using *S. stutzeri* SBB1 would be recommendable for further industrial scale studies.

### 3.7. The Macroalgal Synthesis of Carbon-Dot (CD)

**Figure 15:**
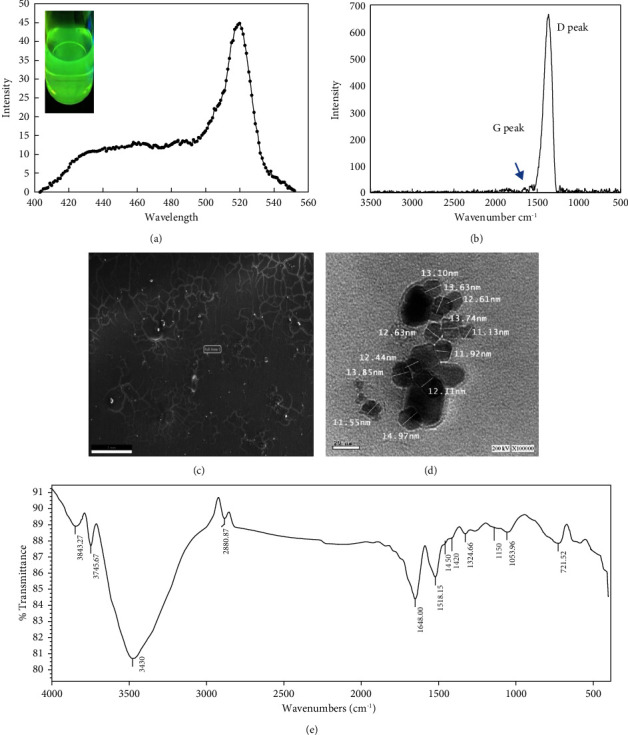
PL spectrum (a), Raman spectrum (b), FESEM image (c), HRTEM image (d), and FTIR spectrum (e) of the algal-synthesized C-dots.

The green synthesis of the CD was done using the aqueous extract of spent waste biomass of *S. latifolium*, disposed of from the bioethanol production process, without using any chemical oxidizing agents. The obtained clear yellowish suspension of CD displayed strong bright green fluorescence under UV light-ʎ_365nm_ (Figure 15(a)). Mewada et al. [126] reported a similar observation. That suggests its suitability for being used in cellular imaging [126]. According to Mewada et al. [126], the recombination of electron-hole pairs from impurity atoms and oxygen-bearing functional groups may be the reason for the CD-fluorescence. The EDX analysis revealed the presence of O, Na, and N, besides C, of approximately 33.76 ± 0.05%, 7.28 ± 0.05%, 4.24 ± 0.05%, and 54.72 ± 0.05% (w/w), respectively, which coincides with the previously reported EDX of green-synthesized CD using *Osmanthus fragrans* flowers [127].

The PL spectrum of the *S. latifolium*-synthesized CD (Figure 15(a)) recorded maximum emission intensity at 520 nm, which matches that reported by Bhattacharya et al. [128].

The Raman spectrum (Figure 15(b)) depicts one main sharp band (1365 cm^−1^), which expresses the D-band. The predominance of D-band over G-band has been previously reported for the green-synthesized CD and indicates functionalized CD with an unassertive graphitic structure [129, 130]. Primarily, D- and G-bands are linked with the vibrational form of sp^3^ and sp^2^ hybridized carbon atoms and denoted for disorder and graphitic band, respectively [130].

The FESEM (Figure 15(c)) and HRTEM (Figure 15(d)) analyses proved the predominance of monodispersed spherical-shaped CS with a narrow average size distribution ranging between 11 nm and 15 nm. According to Hoan et al. [131] and Eskalen et al. [132], the apparent mesh structures of CD in the HRTEM image (Figure 15(d)) would indicate amorphous CD.

The FTIR spectrum (Figure 15(e)) shows an absorption band at 2800 cm^−1^, which can be assigned to -C-H stretching of the methyl or methylene groups associated with the aliphatic hydrocarbons present in the *S. latifolium* extract. The two bands at 3843 cm^−1^ and 3745 cm^−1^ might be due to the O-H stretching of phenolic -OH and -COOH, respectively. The peak around 3430 cm^−1^ might be related to the -NH of amine groups in the *S. latifolium* aqueous extract. The absorption band at 1648 cm^−1^ is due to -C=O, while the vibrational band of -COOH appears around 1324 cm^−1^. The vibrational bands of–C-N and N-OH appear around 1450 cm^−1^ and 1425 cm^−1^, respectively. The peak at 1518 cm^−1^ might be related to -C=C- stretching of aromatics.

The peak around 1053 cm^−1^ might be related to the -C-O-C- linkages, while the peak around 1150 cm^−1^ might be attributed to the stretching band of C-N. The aforementioned functional groups would confirm the carbon dots with aqueous-soluble nature [126]. The FTIR analysis confirmed the EDX analysis, and according to Wu et al. [133] and Lai et al. [134], the presence of the hydrophilic amino, carbonyl, and hydroxyl functional groups enhances the CD water solubility. Moreover, according to Mewada et al. [126], the presence of methyl and hydroxyl groups in CD is also advantageous, as they can serve as linkers for the attachment of therapeutic moieties, such as medications, for specific delivery to diseased cells.

The green-synthesized CD proved to have efficient biocidal activity against different pathogenic microorganisms (Table 8). It expressed higher biocidal activity against the pathogenic fungus *Aspergillus niger* ATCC 16404 and the yeast *Candida albicans* IMRU 3669, relative to the standard metronidazole (100 µg/mL). It recorded an activity index of approximately 1.14 ± 0.01 and 1.33 ± 0.02, with a statistical significant difference from the reference antibiotic of *p* = 0.001 and *p* = 0.0005, at the 95% confidence level, respectively. It is worth mentioning that its activity index against the studied pathogenic fungus and yeast strains is statistically higher than that recorded against the studied pathogenic Gram-positive and Gram-negative bacteria (*p* < 0.0001). Yet, it is still expressing a sufficient biocidal activity against the studied Gram-positive and Gram-negative pathogenic bacteria as compared to the standard antibiotic streptomycin (50 *µ*g/mL). The green-synthesized CD showed a statistically higher biocidal activity on the Gram-negative *Pseudomonas putida* ATCC 10145 relative to that on *Escherichia coli* ATCC 23282 (*p* < 0.0001). Nevertheless, it recorded an activity index of approximately 0.9 ± 0.01 and 0.71 ± 0.03, with a statistical significant difference from the reference antibiotic of *p* = 0.001 and *p* = 0.0003, at the 95% confidence level, respectively. The green-synthesized C-dot expressed a slightly higher biocidal activity against the pathogenic Gram-positive *Bacillus subtilis* ATCC 6633 than that expressed against the pathogenic Gram-positive *Staphylococcus aureus* ATCC 35556 (*p* = 0.001). That recorded a corresponding activity index of approximately 0.78 ± 0.02 and 0.68 ± 0.07, with a statistical significant difference from the reference antibiotic of *p* = 0.0007 and *p* = 0.0002, at the 95% confidence level, respectively. The tabulated antimicrobial efficiency of *S. latifolium* CD (Table 8) is better than that reported for pomegranate peel-based CD [135].

The antimicrobial activity of CD has been previously reported by Dong et al. [136], and it is attributed to the formation of reactive oxygen species (ROS), leading to disruption in bacterial cell walls, damaging the protein, RNA, and DNA, inhibiting some gene expressions, causing leakage of the cytoplasmic contents, and cellular inactivation [137, 138]. However, to the best of our knowledge, it is the first record for the biocidal activity of seaweed-based carbon-dot.

### 3.8. The Attained Valued Merchandises and Their Recommended Conceivable Uses

The main obstacle in utilizing brown macroalgae for the production of bioethanol is the presence of mannitol and alginate. However, this research overcomes such an obstacle by isolating fungal strains capable of hydrolyzing and saccharifying different polysaccharides of *Sargassum latifolium*, in addition to bacteria capable of fermenting pentoses, hexoses, and mannitol. Those yielded approximately 0.32 ± 0.002–0.43 ± 0.002 g fermentable sugars/g algal biomass and 0.25 ± 0.014–0.37 ± 0.02 g ethanol/g algal biomass, respectively. Further work is undergoing now for optimization of the fungal hydrolysis and saccharification of *S. Latifolium* and bioethanol fermentation processes using the obtained microbial isolates to maximize the yield of fermentable sugars and bioethanol.

*S. Latifolium* is found to be an excellent source of natural pigments, antioxidants, carbohydrates, lipids, proteins, fibers, and inorganic minerals, as well as other valuable biopolymers, which are alginate and cellulose. This recommends the implementation of worthwhile biorefinery for green and sustainable products based on marine brown macroalgal biomass (for example, *S. Latifolium*). That would consequently create a new market for the clean and sustainable bio-based blue economy in Egypt, which is expected to be very promising and strategic as macroalgae, its main feedstock, require only carbon dioxide, sunlight, and seawater for growth and proliferation. Moreover, the *S. latifolium* biomass itself can be applied for the production of solid and liquid biofuels. It can also be used in the organic fertilizers and animal fodder industries. Furthermore, it acts as a source of pure alginate, which can be used as stabilizers in the food, beverage, pharmaceutical, and textile industries. Alginate can also be applied for mulching, wastewater treatment, and in many bioprocesses and biomedical applications. It can be used in biofuels, paper, pharmaceutical, and food industries due to its cellulose content.

In addition, the suggested solid-state fungal hydrolysis and saccharification followed by separate bioethanol fermentation and the fully integrated process for extraction of value-added products are completely green and sustainable and do not require any toxic chemicals. They use less energy and water, produce fewer waste effluents, and run under mild operating conditions. Furthermore, the advocated valorization of the spent waste *S. latifolium* into the C-dot would also lower the process waste, decrease the overall cost of the process, and make it more feasible. Since the C-dot has many applications in forensics, imaging, sensors, herbicide detection, heavy metal detection, drug delivery, antioxidants, photocatalysis, optoelectronics, and biocides against pathogenic microorganisms.

Therefore, rather than being viewed as polluting waste, *S. latifolium* can be considered a resource for cleaner production, leading to a net reduction in emissions and enrichment in the industrial sector and economy. Yet, it is worth knowing that the exact cost of *S. Latifolium*-derived merchandise is not yet recognized, and large-scale economic analysis is currently under investigation. However, the results of this study reduce the detrimental effects of harmful macroalgal blooms and beach piles on tourism, the environment, and the economy by maximizing biomass conversion and preventing the spread of invasive macroalgal biomass along the Egyptian Red Sea shorelines.

## 4. Conclusion

This study showed that the readily available massively proliferated brown macroalgae along the Egyptian Red Sea shorelines, *Sargassum latifolium*, which causes eutrophication problems, damages and deteriorates coral reefs, negatively affects aquatic ecosystems and biodiversity, and harms shorelines, can operate as a win-win key along the entire production chain process for a number of energy, industrial, environmental, and economic challenges. Nevertheless, much more research is required to evaluate the commercial and practical viability of the advocated blue-based technology. This is because a lot of variables will affect it, such as the harvesting season and costs, sustainability and feedstock processing, product marketability, and how well they work in other low-carbon footprint industries.

## Figures and Tables

**Table 1:** Water analysis for the collected *S. latifolium* biomass-associated water.

Physical and chemical properties					
Total dissolved solids (TDS)	35298.0 mg/L		Density @ 60 F	1.02847 g/mL	
Salinity (as NaCl)	39475.9 mg/L		Specific gravity	1.02949	
Alkalinity (as CaCO_3_)	128.3 mg/L		pH @ 25°C	7.69	
Total hardness (as CaCO_3_)	6280.2 mg/L		Conductivity	5.55 × 10^−2^ mohs/cm @ 25°C	
BOD	48.4 mg/L		Resistivity	0.1802 ohm-m @ 25°C	
COD	56.3 mg/L		Oil content	11.85 mg/L	

Inorganic chemical constituents					
Cation	mg/L	meq/L	Anion	mg/L	meq/L

Lithium	0.13	0.019	Fluoride	165.60	8.717
Sodium	10918.77	474.748	Chloride	19076.32	537.380
Ammonium	22.55	1.250	Bromide	487.56	6.104
Potassium	352.67	9.021	Nitrate	233.04	3.759
Magnesium	1257.64	103.491	Nitrite	280.01	6.074
Calcium	441.02	22.007	Phosphate	Nil	Nil
Strontium	Nil	Nil	Sulfate	1859.93	38.742
Barium	46.27	0.674	Hydroxide	Nil	Nil
Iron	Nil	Nil	Carbonate	Nil	Nil
Copper	Nil	Nil	Bicarbonate	156.47	2.564

**Table 2:** Algal biomass analysis.

Proximate analysis						
Moisture content %	Volatile content %	Fixed carbon %		Ash content %	Calorific value MJ/kg	
9.2 ± 0.3	66 ± 0.5	0.8 ± 0.05		24 ± 0.5	14.82 ± 0.5	

Biochemical analysis %						
Hemicellulose	Cellulose		Lignin		Ash	

25 ± 0.5	15.5 ± 0.5		8.2 ± 0.5		24 ± 0.5	

Nutritional composition						
Moisture content	Protein content	Fiber content	Carbohydrate content		Lipid content	Ash content
Wt.% (w:w)						

9.2 ± 0.3	15.94 ± 0.31	7.98 ± 0.5	41.5 ± 0.5		1.38 ± 0.05	24 ± 0.5

**Table 3:** Mineral composition of the algal biomass.

	Macronutrients				Micronutrients				Undesired heavy metals						
	P	K	Ca	Mg	Fe	Zn	Mn	Cu	Ni	Cd	Cr	Pb	As	Hg	Co
	mg/kg				mg/kg				mg/kg						
*S. latifolium*	41.7 ± 0.05	208 ± 0.05	214.4 ± 0.05	44.36 ± 0.05	12.63 ± 0.05	24.53 ± 0.05	7.55 ± 0.05	0.24 ± 0.05	0.3021 ± 0.05	0.31 ± 0.05	0.0292 ± 0.05	0.0232 ± 0.05	0.0002 ± 0.0001	0.0002 ± 0.0001	0.325 ± 0.05
Egyptian organic fertilizer standard 8079/2017	—	—	—	—	—	Max 300	—	Max 100	Max 180	Max 5	Max 300	Max 300	—	Max 4	Max 100
Solid biofuels ISO 17225-1	—	—	—	—	—	≤100		≤20	≤10	≤0.5	≤50	≤10	≤1	—	—

**Table 4:** Physicochemical scrutiny of the algal biomass relative to the Egyptian organic fertilizer standard.

Parameter	*Sargassum latifolium*	Egyptian organic fertilizer standard (8079/2017)
Electrical conductivity dS/m	3.37 ± 0.05	6–10
Total dissolved solids (mg/L)	2157 ± 31.8	3840–6400
Organic C %	29.61 ± 0.05	Min 15
Organic matter %	50.92 ± 0.1	Min 18
Moisture content %	9.2 ± 0.3	Max 70
Total nitrogen %	2.55 ± 0.05	Min 0.28
P_2_O_5_ %	0.955 ± 0.01	Min 0.8
K_2_O %	2.496 ± 0.01	Min 0.8
N + P_2_O_5_ + K_2_O %	6.00 ± 0.07	Min 4
C/N	11.61 ± 0.2	18–22 : 1
pH	7.5 ± 0.2	6–8

**Table 5:** The performance of fungal isolates on different polysaccharides and algal biomass.

Fungal code	Fungal isolate		Growth efficiency on different algal polysaccharides		
26	*A. galapagensis* SBWF1		Alginate ≈ cellulose ≈ xylan		
27	*M. hiemalis* SBWF2		Cellulose ≈ xylan > alginate		
28	*P. oxalicum* SBWF3		Alginate ≈ cellulose ≈ xylan		

Hydrolysis and saccharification of *S. latifolium* biomass by the obtained fungal isolates					
Fungal code	Fungal isolate	Produced TRS mg/L	Yield mg/g	Saccharification %	Conversion %

26	*A. galapagensis* SBWF1	1587.4 ± 2.43	317.48 ± 1.75	70.55 ± 0.11	31.75 ± 0.17
27	*M. hiemalis* SBWF2	1684.22 ± 2.98	336.84 ± 1.3	74.85 ± 0.14	33.68 ± 0.13
28	*P. oxalicum* SBWF3	2141.55 ± 2.20	428.62 ± 1.5	95.18 ± 0.10	42.86 ± 0.15
−ve control		100 ± 2.00	20 ± 0.5	4.44 ± 0.09	2 ± 0.05

**Table 6:** The capacities of the bacterial and yeast isolates to utilize different sugars.

Microbial isolates	Mannitol	Lactose	Sucrose	Xylose	Mannose	Fructose	Maltose	Galactose	Glucose	Arabinose
*C. tropicalis* SBY1	++	++	++	++	++	++	++	++	++	++
*S. stutzeri* SBB1	++	++	+++	+++	+++	+++	+++	+++	+++	+++

**Table 7:** The fermentation capabilities of the isolated bacteria and yeast.

Fermentation capabilities on a mixture of standard sugars					
Microbial isolates	Residual sugars mg/L			Bioethanol g/L	Bioethanol yield g ethanol/g sugars
	Glucose	Xylose	Mannitol		
−ve control	2000	2000	2000	Nil	Nil
*C. tropicalis* SBY1	3	1343	1550	2.33 ± 0.035	0.39 ± 0.014
*S. stutzeri* SBB1	2	333	125	4.38 ± 0.212	0.73 ± 0.029

Bioethanol production from *S. latifolium*					
Fungal hydrolyzates	Produced TRS mg/L	*Stutzerimonas stutzeri* strain SBB1			
		Bioethanol g/L	Actual bioethanol yield g ethanol/g TRS	Bioethanol conversion yield	Bioethanol yield g ethanol/g algal biomass

*A. galapagensis* SBWF1	1587.4 ± 0.43	1.25 ± 0.035	0.79 ± 0.007	79%±0.70	0.25 ± 0.014
*M. hiemalis* SBWF2	1682.11 ± 2.98	1.30 ± 0.014	0.77 ± 0.007	77%±0.70	0.26 ± 0.018
*P. oxalicum* SBWF3	2141.55 ± 2.2	1.85 ± 0.048	0.86 ± 0.007	86%±0.70	0.37 ± 0.020

**Table 8:** The antimicrobial activity of the algal-synthesized C-dot.

Compound ID	Tested microorganisms					
	*B. subtilis* ATCC 6633	*S. aureus* ATCC 35556	*E. coli* ATCC 23282	*P. putida* ATCC 10145	*C. albicans* IMRU 3669	*A. niger* ATCC 16404
C-dot	25 ± 0.45	23 ± 0.36	17 ± 0.29	27 ± 0.48	28 ± 0.35	25 ± 0.29
Reference antibiotic	32 ± 0.7	34 ± 0.7	24 ± 0.44	30 ± 0.66	21 ± 0.52	22 ± 0.44
Activity index	0.78 ± 0.02	0.68 ± 0.07	0.71 ± 0.03	0.9 ± 0.01	1.33 ± 0.02	1.14 ± 0.01

## Data Availability

Data are available from the corresponding author upon request.
